# Bright single-photon skyrmion sources in bullseye cavities

**DOI:** 10.1515/nanoph-2025-0488

**Published:** 2025-11-10

**Authors:** Jiantao Ma, Shunfa Liu, Chengjie Lu, Ying Yu, Bo Chen, Jin Liu

**Affiliations:** State Key Laboratory of Optoelectronic Materials and Technologies, School of Electronics and Information Technology, School of Physics, 26469 Sun Yat-Sen University, Guangzhou, 510275, China; Exeter College, University of Oxford, Oxford, OX1 3DP, UK; Quantum Science Center of Guangdong-Hong Kong-Macao Greater Bay Area, Shenzhen, 518102, China

**Keywords:** optical skyrmions, bullseye cavity, single-photon sources

## Abstract

Optical skyrmions, as structured light fields endowed with discrete topological numbers, open new opportunities for high-density encoding, robust information transport, and quantum light–matter interactions. However, most existing skyrmion generators rely on complex or bulky systems, hindering their application in scalable on-chip quantum technologies. Here, we propose a nanophotonic scheme based on semiconductor cavity quantum electrodynamics, whereby a circularly polarized quantum emitter is coupled to a concentric bullseye resonator. This configuration enables the efficient generation of single-photon Stokes vector skyrmions at subwavelength scales, as well as their high-order extensions. By exciting single-photon sources at different positions, the skyrmion number can be continuously switched between +2 and −2, while higher-order states are accessible by tuning the radius of cavity’s center disc. This strategy couples the topological dimension of skyrmions with quantum states, laying the groundwork for quantum skyrmions in on-chip topological keying and quantum readout. Our work provides a practical device architecture for integrated nanophotonic quantum topological state platforms, offering a new paradigm for topologically protected quantum communications and on-chip quantum information processing.

## 1 Introduction

Topological photonics has garnered increasingly interest in optical skyrmions, whose discrete integer-valued topological invariants and robustness against smooth perturbations endow them with particle-like stability [1], [2], [3]. Optical skyrmions can be realized across diverse three-dimensional (3D) vector fields, encompassing electric and magnetic fields [4], [5], [6], spin angular momentum [7], [8], [9], [10], polarization Stokes vectors [11], [12], [13], [14], [15], [16] and so on. Such vector structured light, with its rich topological textures, provides new degrees of freedom beyond amplitude and phase for light manipulation and information encoding [3], [17]. Early pioneering work established feasibility and nanoscale vectorial features for skyrmionic lattices in evanescent electromagnetic waves of surface plasmon polaritons [4] and deep-subwavelength spin skyrmions formed by spin–orbit coupling in confined vortex fields [7]. These breakthroughs paved the way for the field to expand into other regimes, including Stokes-vector skyrmions in paraxial vector beams [13] and spatiotemporal electromagnetic skyrmions in supertoroidal pulses [6], further broadening the methods for manipulating the topological properties of light.

However, most current skyrmion generation methods rely on bulk optics [6], [14], [15], [18], [19], [20], [21], [22], [23], such as interferometric superpositions and spatial light modulators [14], [19], [23], to synthesize orthogonally polarized modes with tailored orbital angular momentum (OAM), which inherently restricts system stability and scalability. While some progress has been made toward integration, including skyrmions in microcavity through spin–orbit interaction [16], [24], [25], compact waveplates [26], [27], and meta-platforms [28], [29], [30], [31], the overall development of scalable and practical devices remains limited. Moreover, a significant quantum milestone was achieved with the creation of non-local skyrmions as entangled states of light [32], [33], unveiling profound connections between topology and quantum entanglement and opening avenues for topology-enhanced quantum information. Further advancing into the integrated quantum realm, a semiconductor cavity quantum electrodynamics (cQED) platform has directly generated single-photon skyrmions [34], marking a critical transition from bulk optics to nanophotonics and from classical to quantum system. However, the Gaussian microcavity supports only low-order skyrmions and does not offer a straightforward method for on-chip tuning of the topological texture. The single-photon generation rate and extraction efficiency also remain far from optimal.

Despite these promising advances, a platform that simultaneously offers high efficiency and full compatibility with quantum operations has yet to be developed. To address this gap, we propose an integrated nanophotonic scheme that couples a circularly polarized single-photon emitter to a concentric bullseye resonator. This designed microcavity structure, comprising a circular Bragg grating (CBG), a dielectric spacer and a metallic mirror, provides strong lateral confinement and highly efficient vertical out-coupling [35], [36]. By placing a circular dipole at the cavity’s center, the right- and left-handed circularly polarized (RCP and LCP) components of the excited cavity mode carry distinct OAM orders, whose superposition generates a skyrmionic vector polarization field with a quantized skyrmion number. Leveraging cavity-enhanced spin–orbit coupling and mode engineering, this system synthesizes Stokes vector skyrmions and thus enables the emission of single-photon skyrmions at subwavelength scales. Our optimization at 925 nm achieves a Purcell factor of approximately 32 with an extraction efficiency exceeding 98 %, ensuring bright single-photon operation and high-fidelity Stokes-vector textures. Besides, the emitter’s chirality and slight displacement enable polarity reversal and continuous switching of the skyrmion number between +2 and −2. Furthermore, by tuning the radius of the cavity’s center disc to access higher-order radial modes, the same device architecture can generate more complex skyrmions [37], [38], such as higher-order 7*π* and 11*π* skyrmions. This capability bridges fundamental and higher-order topologies on a single, unified platform, paving the way for robust quantum topological photonics [39], [40], [41], [42].

## 2 Results and discussion

**Figure 1:**
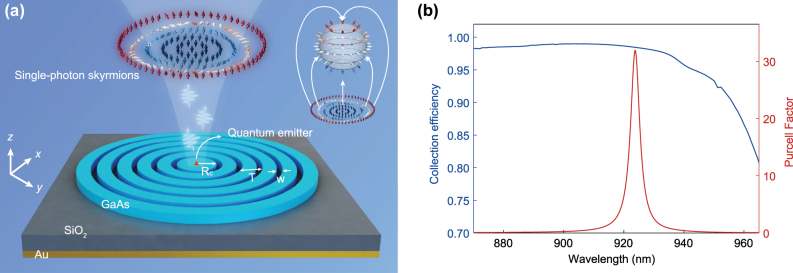
Single-photon skyrmions emitted from a quantum emitter coupled to a bullseye resonator. (a) Schematics of the device for single-photon skyrmions generation. The single photons emitted from the quantum emitter are coupled to the cavity modes of the bullseye resonator and radiated to free space with skyrmionic texture. Inset: Mapping from the polarization vector field to a unit Poincaré sphere. (b) Numerical simulations of the purcell factor and the extraction efficiency of the single-photon source. By optimizing the structure of the resonator, a Purcell factor of ∼32 and collection efficiency over 98 % are obtained at the emitting wavelength near 925 nm. The parameters of the optimized bullseye resonator are: central disc radius *Rc* = 360 nm, circular grating period *T* = 360 nm, trench width *w* = 110 nm. The thickness of GaAs layer is 160 nm and the thickness of the SiO_2_ layer is 250 nm. In Figure 1(a) and throughout this paper, the orientation of the vector arrows represents different in-plane spin azimuths of the Stokes vectors, and the color of the vector arrows corresponds to out-of-plane components of the Stokes vectors.

The proposed device for generating single-photon skyrmions is schematically illustrated in Figure 1(a). A circularly polarized quantum emitter is positioned at the center of a bullseye resonator. Through its interaction with the bullseye cavity, the photons emitted by the quantum emitter are efficiently funneled into free space while acquiring skyrmionic polarization texture. The bullseye resonator consists of a CBG with thickness of 160 nm, a 250 nm SiO_2_ spacer, and a 100 nm high-reflectivity Au layer. The CBG, formed by a series of concentric trenches with increasing diameters, confines the in-plane propagating photons toward the cavity center. The SiO_2_ spacer and Au layer together serve as a bottom mirror, reflecting downward-emitted photons back into the cavity and thereby enabling highly efficient vertical emission. To maximize the device performance, we optimize the bullseye structure for a quantum emitter operating at a wavelength of 925 nm. Using the finite-difference time-domain (FDTD) method, we determined the optimal parameters to be a central disc radius (*Rc*) of 360 nm, a circular grating period (*T*) of 360 nm, and a trench width (*w*) of 110 nm. To maximize the Purcell enhancement, the single-photon emitter is positioned at the vertical center of the 160 nm GaAs layer. As shown in Figure 1(b), this optimized subwavelength cavity achieves a Purcell factor of ∼32 at the 925 nm emission wavelength, signifying a strong enhancement of the spontaneous emission rate. Furthermore, with a photon extraction efficiency exceeding 98 %, the device holds great potential for use as a bright single-photon source.

To achieve efficient emission of single-photon skyrmions from the bullseye cavity, a circularly polarized dipole serves as the quantum emitter to excite the skyrmionic mode. In the concentric bullseye resonator, the central disk can be approximately described by the Helmholtz equation [43]. The Stokes vector ***S*** = (*S*_1_, *S*_2_, *S*_3_) can represent an arbitrary state of polarization as points on the surface of the unit-radius sphere known as the Poincare sphere [14], [44]. When a circularly polarized single-photon emitter is located at the center of the concentric bullseye resonator, it excites the transverse zeroth- and second-order Bessel modes. The coupling coefficients for this excitation are proportional to the local field overlap and polarization matching.

**Figure 2:**
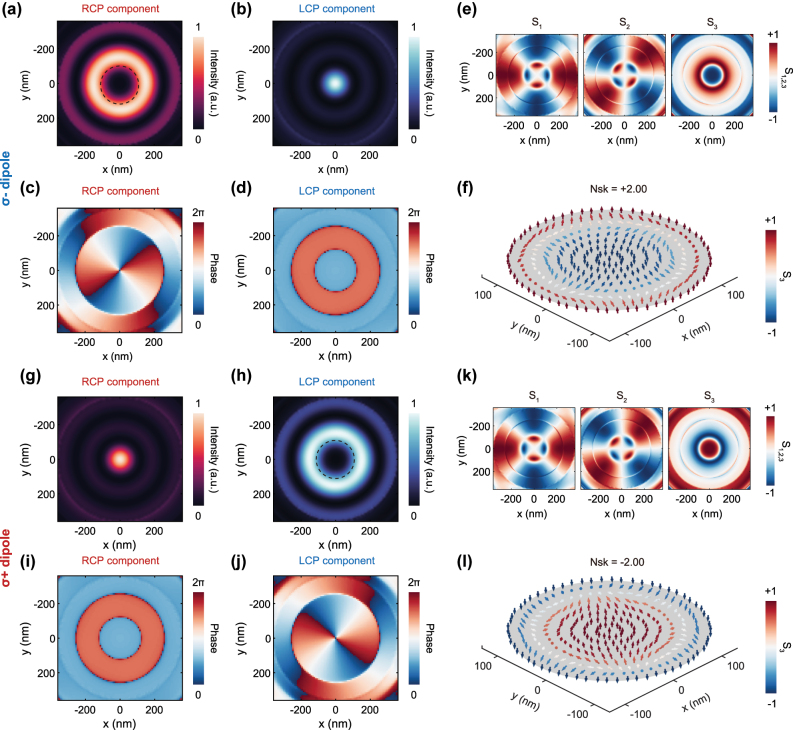
Simulated skyrmionic polarization textures when a circularly polarized quantum emitter located in the center of the bullseye resonator. (a–f) Simulations of a LCP quantum emitter in cavity. The intensity profiles (a, b) and the phase distributions (c, d) indicate that the RCP and LCP component of the emitted photons carry OAM with order *l* = −2 and 0, respectively. According to the extracted Stokes parameters (e), the normalized Stokes vectors distribution with skyrmion number *N*_sk_ = +2.00 is obtained (f). (g–l) Simulations of a RCP quantum emitter in cavity. The intensity profiles (g, h) and the phase distributions (i, j) indicate that the RCP and LCP component of the emitted photons carry OAM with order *l* = 0 and +2. According to the extracted Stokes parameters (*k*), the normalized Stokes vectors distribution with skyrmion number *N*_sk_ = −2.00 is obtained (l). The skyrmion areas are enclosed within the dashed black lines in (a) and (h).

We first examine the case of a LCP dipole placed at the center of the cavity. The resulting RCP and LCP components of the emitted photons are shown in Figure 2(a)–(d). The simulation results reveal that the RCP component exhibits a doughnut-like intensity profiles with a helical phase, corresponding to an OAM mode with a topological charge of *l* = +2, whereas the LCP component displays a Gaussian-like mode without OAM. This simulation results further indicate that the central lobes of the zeroth- and second-order Bessel modes form the effective regions. The coexistence of these two components, each with a different OAM order, enables the formation of a vector polarization field with a skyrmionic texture. To confirm the skyrmionic texture, we calculated the normalized Stokes vector, ***S*** = (*S*_1_, *S*_2_, *S*_3_), shown in Figure 2(e), and visualized the corresponding vector field with an arrow plot. Arbitrary polarization states can be represented by the normalized Stokes vector, with the in-plane spin azimuth arctan (*S*_1_/*S*_2_) (and out-of-plane component *S*_3_) mapped to the in-plane orientation (and color) of the vector arrow. As seen in Figure 2(f), the arrows flip upward (from the center to the periphery) along the radial direction while simultaneously completing two full rotations azimuthally. To characterize the topological properties of the single-photon skyrmion sources, we calculate the skyrmion number of the quantum skyrmions, which is defined as [45], [46]

(1) $$N_{\text{sk}}=\frac{1}{4\pi }\underset{U}{\iint }S\cdot \left(\frac{\partial S}{\partial x}\times \frac{\partial S}{\partial y}\right)dxdy$$

where ***S*** (*x*, *y*) represents the vector field to construct a skyrmion and *U* denotes the region to confine the skyrmion. By integrating over the vector field within the skyrmion region (marked by the black dashed line in Figure 2(a)), we calculate a topological invariant with skyrmion numbers of +2. The distinct vectorial winding and the skyrmion number confirms the generation of single-photon skyrmions. To analyze the far-field characteristics of the emitted single-photon skyrmions, it is essential that the topology is preserved during propagation from the near-field to the far-field.

In contrast, placing an RCP dipole at the cavity center generates single-photon skyrmions with the opposite polarity. In this configuration, the LCP component carries an OAM of *l* = −2 (Figure 2(g) and (i)), while the RCP component has no OAM (Figure 2(h) and (j)). This inverts the polarity of the resulting skyrmion, which is confirmed by the sign reversal of the Stokes parameter *S*_3_ (Figure 2(k)). Along the radial direction, the arrows flip from pointing up at the center to down at the edge (Figure 2(l)). This full inversion corresponds to a skyrmion number of *N*_sk_ = −2. These results demonstrate that single-photon skyrmions can be realized in a bullseye cavity coupled to a circularly polarized quantum emitter. Moreover, the polarity of the generated quantum skyrmions can be deterministically controlled by switching the handedness of the quantum emitter.

**Figure 3:**
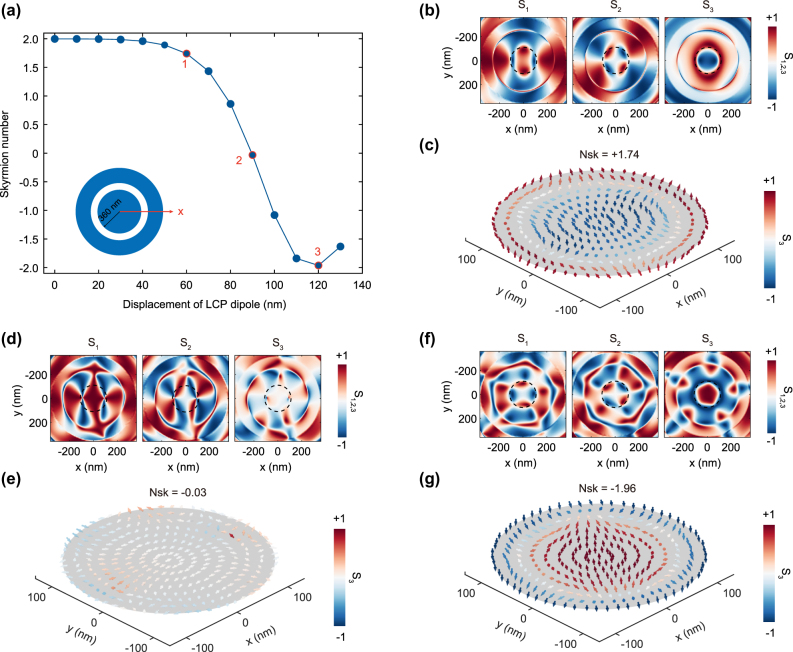
Simulations of the single-photon emissions when a LCP quantum emitter located away from the center of the bullseye resonator. (a) The skyrmion number of the emitted single-photon as a function of the displacement of the LCP quantum emitter along the *x* direction. (b, d, f) The extracted Stokes parameters of the emitted photons when the quantum emitter is displaced by 60 nm, 90 nm and 120 nm, respectively. (c, e, g) The normalized Stokes vectors distributions when the quantum emitter is displaced by 60 nm, 90 nm and 120 nm, respectively. By locating the quantum emitter at the center and the edge of the skyrmion area of the cavity mode, single-photon skyrmions with opposite polarity can be obtained.

In practical fabrication of quantum-emitter-cavity devices, the emitter often deviates from the bullseye cavity center due to unavoidable placement and fabrication errors, making it essential to assess the impact of displacement. We therefore examine how the skyrmion number of the emitted photons varies with dipole offset along the *x* direction. For an LCP dipole placed at successive off-center positions, we extract the Stokes vector field and compute the corresponding skyrmion number in each case. The integration region is fixed to that of the centered reference (identical to Figure 2(a)). As shown in Figure 3(a), the skyrmion number evolves continuously from +2 to −2 as the dipole is moved outward radially. This evolution arises from the superposition of the skyrmion states corresponding to *N*_sk_ = +2 and *N*_sk_ = −2, with their relative contributions varying with the emitter’s displacement.

Three representative cases are illustrated in Figure 3(b)–(g), corresponding to emitter displacements of 60 nm, 90 nm, and 120 nm, respectively. From the results in Figure 2(f) and (l), it can be seen that within the skyrmion vector field itself, the local polarization state deviates from perfect circular polarization when moving slightly away from the skyrmion center. Consequently, when an LCP dipole is displaced to such a position (e.g., 60 nm), it still predominantly excites an LCP-centered skyrmion state (*N*_sk_ = +2), but also weakly excites an RCP-centered skyrmion (*N*_sk_ = −2). This mixing of states reduces the total skyrmion number from its ideal integer value (*N*_sk_ = +1.74), as shown in Figure 3(b) and (c). As the radial displacement increases, the local polarization of the excited field evolves from circular to elliptical and, eventually, to linear, accompanied by a corresponding decrease in skyrmion number. Figure 3(d) and (e) show the case of a 90 nm displacement, the vector field polarization is nearly linear (Figure 3(d) and (e)). Here, the LCP dipole excites both *N*_sk_ = +2 and −2 skyrmion states almost equally, causing the *S*_3_ parameter to approach zero within the skyrmion region. The resulting linear vector field has a skyrmion number near zero. With further displacement, the handedness of the vector field reverses, and its polarization evolves back from linear to elliptical and finally to circular. At a 120 nm displacement, the LCP-dipole predominantly radiates photons with an RCP-centered skyrmionic texture with *N*_sk_ = −2 (Figure 3(f) and (g)). These findings systematically illustrate how the emitted photons’ skyrmionic topology evolves with the quantum emitter’s radial position. Notably, the skyrmion number remains highly stable even when the emitter is shifted from the center of the bullseye cavity by up to 40 nm in the *x*-direction, indicating its robustness against positional perturbations. This robustness is consistent with recent findings on the topological protection of optical skyrmions [47], [48]. This provides theoretical guidance on fabrication tolerances and introduces a practical method for controlling single-photon skyrmion polarity by precisely positioning the emitter.

While the previous results only demonstrate skyrmions with topological charges of ±2, our design can be readily extended to generate higher-order skyrmions with more complex topologies by simply increasing the radius of the cavity’s central disk. By adjusting the bullseye cavity design, we further explore the generation of more complex topological single-photon states in the quantum-emitter-coupled bullseye cavity, such as skyrmionium or *kπ* skyrmion. Unlike a conventional skyrmion, which has a single radial vector reversal, a skyrmionium is a nested texture with an additional flip, creating a 2*π* radial twist and a net skyrmion number of zero. An extension of this, the *kπ*-skyrmion (or target skyrmion), incorporates multiple *π*-twists, enabling fine control over the topological charge.

**Figure 4:**
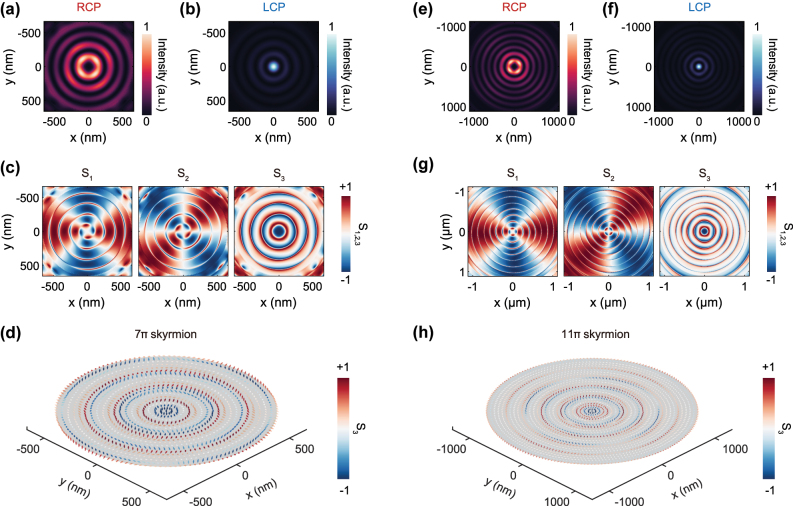
The generation of *kπ-*skyrmions (target skyrmions) by increasing the radius of the central disk in the bullseye resonator. (a–d) Characteristics of simulated 7*π* skyrmions emissions when a LCP quantum emitter located at the center of the bullseye resonator with *Rc* = 660 nm. (e–f) Characteristics of simulated 11*π* skyrmions emissions when a LCP quantum emitter located at the center of the bullseye resonator with *Rc* = 1,120 nm.

To create these states, the cavity needs to support higher-order radial modes that have multiple concentric intensity rings. We achieved this by increasing the radius of the central disc and tuning the cavity resonance to match the quantum emitter wavelength of 925 nm. Two cavity geometries with central disk radii of 660 nm and 1,120 nm were designed, yielding the results shown in Figure 4(a)–(d) and (e)–(h), respectively. In both cases, the RCP and LCP components of the emitted field feature additional concentric rings, which confirms the excitation of higher-order radial cavity modes (Figure 4(a), (b), (e), (f)). The Stokes parameters *S*_1_ and *S*_2_ remain similar to those of a fundamental skyrmion, preserving the azimuthal vector–vortex structure (Figure 4(c) and (g)). However, the *S*_3_ parameter undergoes multiple sign reversals along the radial direction, corresponding to repeated upward-downward flips of the vector field arrows in Figure 4(d) and (h). This confirms the formation of higher-order photonic skyrmion states, specifically 7*π* skyrmions and 11*π* skyrmions, respectively. This result demonstrates that by engineering the cavity geometry, we can systematically control the radial complexity of emitted single-photon skyrmions, opening a promising route toward high-capacity topological encoding for quantum information processing.

## 3 Conclusions

In conclusion, we have proposed and numerically demonstrated an integrated nanophotonic platform for the deterministic generation of single-photon skyrmions. By coupling a circularly polarized quantum emitter to a specially designed bullseye resonator, our scheme achieves bright, efficient single-photon emission with engineered topological properties. Our results show that the chirality of the quantum emitter can deterministically control the polarity of the generated Stokes vector skyrmions, producing integer skyrmion numbers of +2 or −2. Furthermore, we demonstrated that continuous tuning of the skyrmion number between +2 and −2 can be achieved by radially displacing the emitter from the cavity center. This finding not only provides insight into fabrication tolerances but also offers a practical method for manipulating the topological charge. Finally, we have shown that by engineering the cavity geometry to support higher-order radial modes, the same device architecture can be extended to generate more complex topological states, such as 7*π* and 11*π* skyrmions. As a new paradigm for photonic quantum information, single-photon skyrmions are merging the fields of topological photonics and quantum technologies. Their unique properties open up several promising application avenues. The single-photon skyrmions, characterized by a topological number, is resilient to smooth perturbations and environmental noise. Moreover, the rich, non-trivial spin texture of a single-photon skyrmion inherently encodes a high-dimensional state. Furthermore, the ability to generate and control various skyrmionic states, including higher-order topologies like skyrmioniums or *kπ* skyrmions, offers multiple degrees of freedom for encoding information [49]. This work bridges the gap between fundamental and higher-order topological photonics on a unified, integrated platform, opening a pathway toward advanced quantum information processing and high-capacity quantum communications using topological single photons.
